# Four Regional Marine Biodiversity Studies: Approaches and Contributions to Ecosystem-Based Management

**DOI:** 10.1371/journal.pone.0018997

**Published:** 2011-04-29

**Authors:** Sara L. Ellis, Lewis S. Incze, Peter Lawton, Henn Ojaveer, Brian R. MacKenzie, C. Roland Pitcher, Thomas C. Shirley, Margit Eero, John W. Tunnell, Peter J. Doherty, Brad M. Zeller

**Affiliations:** 1 Aquatic Systems Group, University of Southern Maine, Portland, Maine, United States of America; 2 Fisheries and Oceans Canada, St. Andrews Biological Station, St. Andrews, New Brunswick, Canada; 3 Estonian Marine Institute, University of Tartu, Pärnu, Estonia; 4 National Institute for Aquatic Resources, Technical University of Denmark (DTU-Aqua), Charlottenlund, Denmark; 5 Department of Marine Ecology, University of Aarhus, c/o DTU-Aqua, Charlottenlund, Denmark; 6 Department of Biology, Center for Macroecology, Evolution and Climate, University of Copenhagen, Copenhagen, Denmark; 7 Commonwealth Scientific and Industrial Research Organisation Marine and Atmospheric Research, Cleveland, Australia; 8 Harte Research Institute for Gulf of Mexico Studies, Texas A&M University-Corpus Christi, Corpus Christi, Texas, United States of America; 9 Australian Institute of Marine Science, Townsville, Queensland, Australia; 10 Department of Employment, Economic Development and Innovation, Fisheries Queensland, Brisbane, Queensland, Australia; University of Glamorgan, United Kingdom

We compare objectives and approaches of four regional studies of marine biodiversity: Gulf of Maine Area Census of Marine Life, Baltic Sea History of Marine Animal Populations, Great Barrier Reef Seabed Biodiversity Project, and Gulf of Mexico Biodiversity Project. Each program was designed as an “ecosystem” scale but was created independently and executed differently. Each lasted 8 to 10 years, including several years to refine program objectives, raise funding, and develop research networks. All resulted in improved baseline data and in new, or revised, data systems. Each contributed to the creation or evolution of interdisciplinary teams, and to regional, national, or international science-management linkages. To date, there have been differing extents of delivery and use of scientific information to and by management, with greatest integration by the program designed around specific management questions.

We evaluate each research program's relative emphasis on three principal elements of biodiversity organization: composition, structure, and function. This approach is used to analyze existing ecosystem-wide biodiversity knowledge and to assess what is known and where gaps exist. In all four of these systems and studies, there is a relative paucity of investigation on functional elements of biodiversity, when compared with compositional and structural elements. This is symptomatic of the current state of the science. Substantial investment in understanding one or more biodiversity element(s) will allow issues to be addressed in a timely and more integrative fashion. Evaluating research needs and possible approaches across specific elements of biodiversity organization can facilitate planning of future studies and lead to more effective communication between scientists, managers, and stakeholders. Building a general approach that captures how various studies have focused on different biodiversity elements can also contribute to meta-analyses of worldwide experience in scientific research to support ecosystem-based management.

## Introduction

Marine ecosystems provide a wide variety of services, including provision of food, regulation of climate, support via primary production and nutrient recycling, and cultural enrichment [1]. However, many coastal and shelf ecosystems are currently degraded from their earlier states [2]–[5], which compromises the services they can provide. One of the fundamental challenges in marine ecology is to relate the nature and magnitude of ecosystem services to the extent of habitats and communities, the biodiversity that they contain, and the types and levels of disturbance they can endure, because humans will continue to both use and depend on the marine environment [6]. During the past decade, there have been repeated calls for integrated approaches to managing marine resources that consider the entire ecosystem, including humans [1], [6]–[13]. Ecosystem-Based Management (EBM) [10] and Ecosystem Approaches to Management (EAM) [14] have been broadly advocated as terms to reflect this new approach (here we use EBM). Both approaches share the common goal of managing human activities to sustain resources, and they promote conserving biodiversity as a keystone to maintaining ecosystem function and adaptation over long periods of time [6], [15], [16]. To conserve biodiversity, managers require scientific information on the patterns and role of biodiversity in the system they are managing.

**Figure 1:**
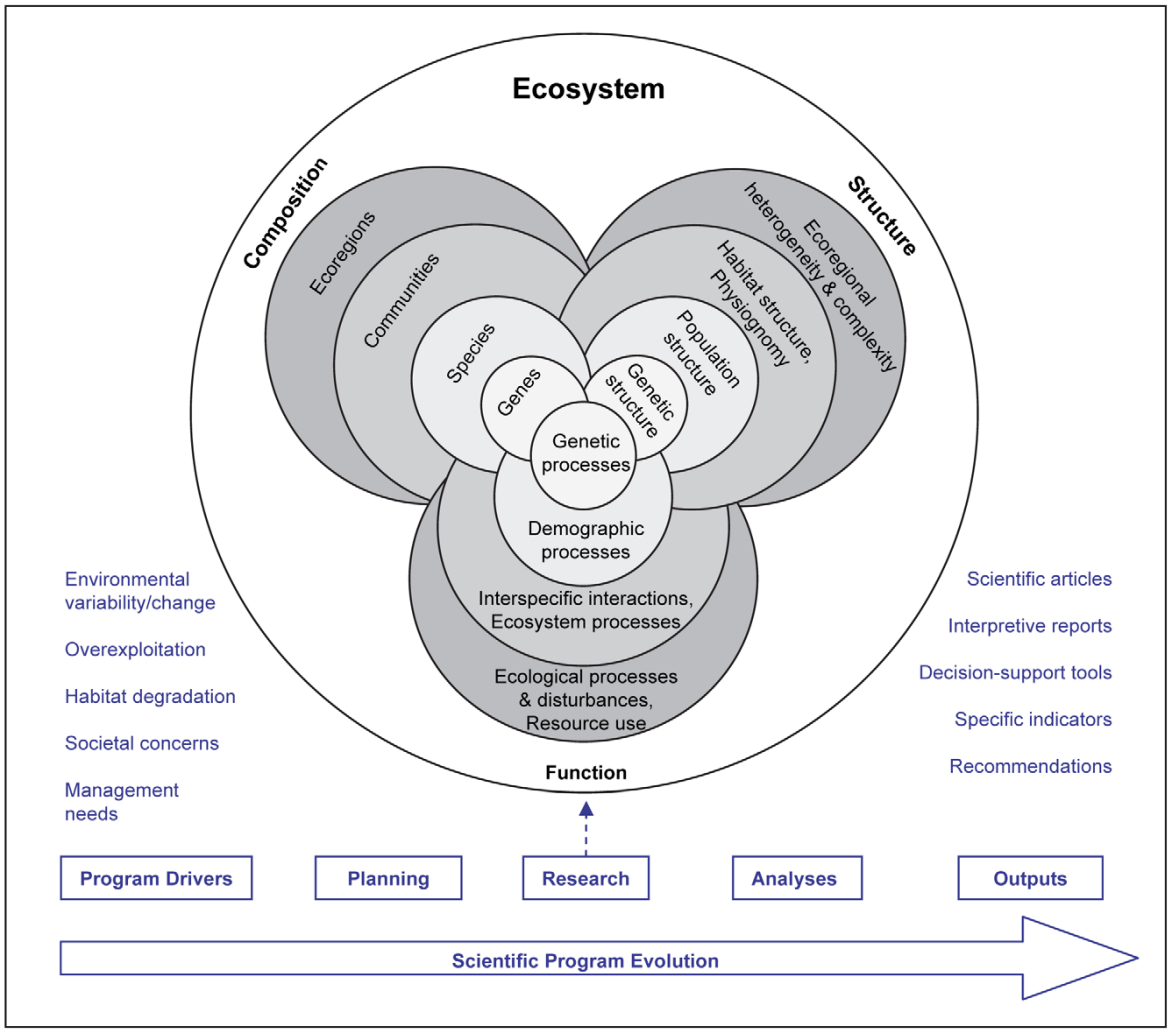
Elements of biodiversity research needed to support ecosystem-based management. Decreasing scales of biodiversity, from ecoregions to genes, are depicted from the outer to inner core of each element. Scientific program evolution is depicted by steps above the horizontal arrow. (Feedback loops for iterative programs are not included.) Examples of program drivers are listed at left. EBM uses insights provided by detailed research rather than the myriad research results themselves. These insights are summarized or integrated as outputs, such as the examples listed at right. (Figure adapted from [20], [21].)

Biodiversity is often considered in terms of species richness, or the number of species in a given area. However, the full spectrum of biodiversity encompasses multiple levels of biological organization, from genetic diversity within populations, to species diversity within communities, to community diversity across landscapes and ecosystems [17], [18]. Biodiversity within an ecosystem can be conceptualized by three principal elements: composition, structure, and function [19]–[21]. These interconnected elements can be represented in a hierarchy that incorporates four nested levels of biological organization and spatial scales ranging from ecoregions to genes (Figure 1). (In this context, “ecoregion” refers to large marine areas characterized by distinctive oceanographic and ecological features that are useful for planning or management [22]–[24]). This classification approach was originally proposed to organize strategies and technical approaches for monitoring biodiversity in terrestrial systems [20], and was subsequently adapted by Cogan and Noji [21] and Cogan et al. [25] to demonstrate how marine biodiversity analysis and habitat mapping might be used to implement EBM. We believe that this approach can serve an even broader purpose by helping to connect basic biodiversity science with the management of ocean space and resources [26].

*Compositional* marine biodiversity elements represent the identity and variety of biodiversity within the system, from ecoregions to genes. Examples include physiographic regions, habitat types, species lists, and genotypes. *Structural* marine biodiversity elements are concerned with the physical organization or patterns of biotic and abiotic variables within the system, e.g., the arrangement, heterogeneity, and complexity of subregions and habitats, structures of populations, and phenotypic expression of genotypes. *Functional* marine biodiversity elements are natural and anthropogenic processes and disturbances that operate at various spatial, ecological, and evolutionary scales to mold biodiversity composition and structure. These can be organized as environmental processes and disturbances (e.g., currents, tides, mixing, resource extraction), interspecific interactions and other ecosystem processes (e.g., predation, competition, disease), demographic processes and life histories (e.g., migration, recruitment, survivorship, behavior) and genetic processes (e.g., mutation, selection, gene flow). It is important to clarify that in this classification approach, functional elements operate *within* the system to affect or modulate biodiversity characteristics and are thus not directly analogous to the “function” that marine biodiversity has in supporting ecosystem services (*sensu* [27], [28]); the latter benefits to human populations are best considered separately. However, human uses of the marine environment, including disturbances to the environment and alteration of populations (through targeted removals and other effects), are considered within the set of functional elements because they can affect biodiversity composition, structure, and function [26]. (For more examples of biodiversity elements see [21] and [26]).

**Figure 2:**
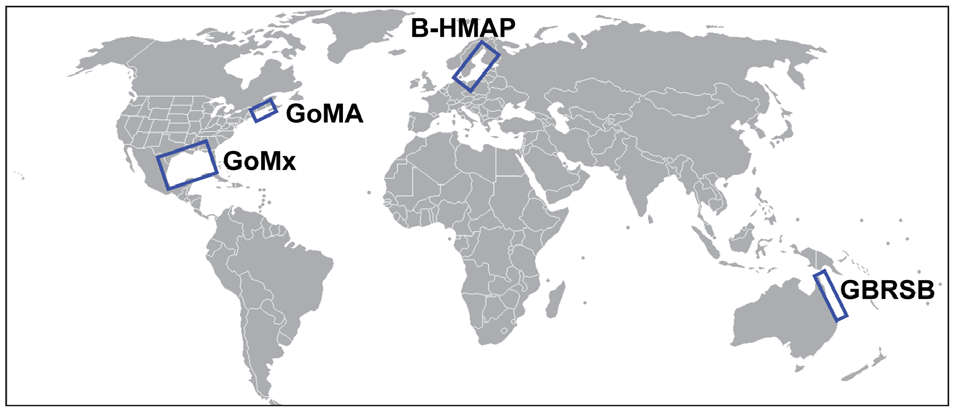
Locations of four regional research programs on marine biodiversity. Baltic Sea History of Marine Animal Populations (B-HMAP), Great Barrier Reef Seabed Biodiversity Project (GBRSB), Gulf of Mexico Biodiversity Assessment (GoMx), and Gulf of Maine Area Census of Marine Life (GoMA). Each of these programs has contributed to the Census of Marine Life [30], [31].

Biodiversity research and monitoring programs can be directed at one or more of these elements and at various spatial or biological scales (Figure 1). Scientists summarize or integrate these insights in outputs such as recommendations and indicators (Figure 1). Managers and practitioners of EBM use insights provided in these outputs, rather than the myriad research results themselves. Ecosystem studies typically join basic science with applied science. Here, we compare the objectives, approaches, achievements, contributions to EBM, and general “lessons learned” from four ecosystem-level studies of marine biodiversity conducted in diverse environments: Gulf of Maine Area Census of Marine Life, Baltic Sea History of Marine Animal Populations, Great Barrier Reef Seabed Biodiversity Project, and Gulf of Mexico Biodiversity Assessment (Figure 2). Each program contributed to the Census of Marine Life, a 10-year scientific initiative to assess and explain the diversity, distribution, and abundance of life in the oceans [29]–[31]. The four systems differ widely in size, species richness (from about 5,000 to more than 15,000 species on a regional basis), and oceanographic characteristics (from tropical to boreal-temperate) (Table 1).

**Table 1:** Comparison of four study regions.

Descriptor	Gulf of Maine Area	Baltic Sea	Great Barrier Reef Shelf	Gulf of Mexico
Size (km^2^)	GoM proper 123,600 Adjacent areas 104,400^1^ Total 228,000^1^	415,000	GBR shelf 210,000	1,510,000
Latitudinal range	40°N to 46°N	53°N to 66°N	10.7°S to 24.5°S	18°N to 30.5°N
Average depth (m)	245^1^	60	42	1,500
Maximum depth (m)	366^2^, 3,000^3^	460	130	>3,800
Average annual primary productivity (g C/m^2^/yr)	270	135^4^	N/A	150–300
Main stressors	Climate change, Fishing, Habitat loss, Coastal development	Climate change, Eutrophication, Pollution, Fishing, Shipping	Climate change, Run-off water quality, Fishing, Shipping, Tourism	Hypoxia, Fishing, Habitat loss, Water quality
Number of named marine species	>5,600	>6,065^5^	>7,000	15,419
Number of bordering countries	2	9	1	3

1 Wolff and Incze, unpublished.

2 Inside Gulf of Maine proper.

3 At seaward edge of Bear Seamount (Wolff and Incze, unpublished).

4 [174].

5 Total species richness [89].

## Gulf of Maine Area Census of Marine Life: Case Study

### Regional description

#### Environmental context

**Figure 3:**
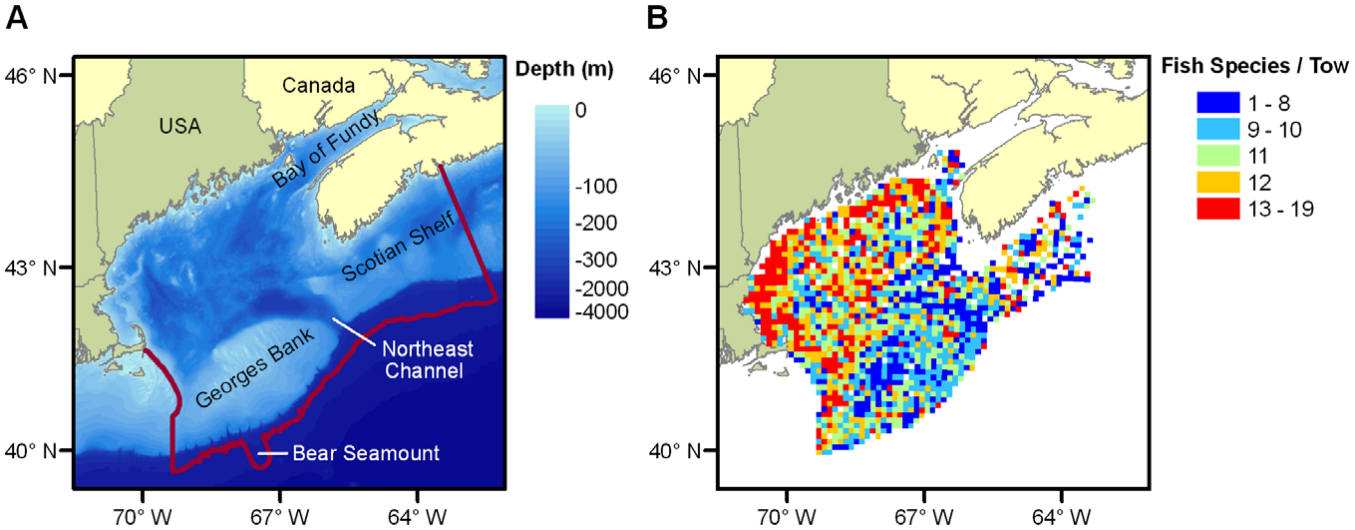
Map and representative data from the Gulf of Maine Area Census of Marine Life. (A) Study area, with marine borders outlined in red. (B) Sample diversity of fish in the Gulf of Maine Area (number of species per tow per 10-by-10 km cell), based on spring trawl surveys of the Northeast Fisheries Science Center, 1968–2008. Spring surveys took place between March and June (90% in March & April) and include 7,556 tows. Samples included 143 species of fish, including 11 elasmobranchs. Groupings are quintiles of the frequency distribution of the samples. There is no correlation between sample diversity and the number of tows per cell.

The Gulf of Maine is a highly productive marginal sea [32], [33] centered at approximately 45°N latitude on the eastern coast of North America. The Gulf of Maine Area Census of Marine Life (GoMA) included all of the Gulf of Maine, Georges Bank, the western half of the Scotian Shelf, the adjacent continental slope to 2,000 m depth, and Bear Seamount, which is located on the slope (Figure 3A) [34]. Shallow banks and shoals along the outer periphery of the Gulf restrict exchanges with the open Atlantic Ocean. Surface circulation is generally counterclockwise around the Gulf, and the majority of water exits around the northern end of Georges Bank [35]–[37]. In the northern Gulf and over many of the offshore banks and shoals, strong tidal mixing creates unstratified or only weakly stratified conditions year-round [38], whereas elsewhere there is strong seasonal stratification.

The Gulf of Maine area has lower biodiversity than many other parts of the world [39] and is generally less diverse than the northeastern Atlantic [40], [41] or the remainder of the U.S. east coast to the south [40], [42]. Cape Cod, which partially defines the western boundary of the study area (Figure 3A), is generally recognized as the transition between the southern Virginian and the northern Acadian biogeographic provinces/ecoregions [24], [43]–[45]. The Gulf is an area of steep latitudinal temperature change, and ranges of many species may have shifted northward in response to the recent regional warming trend of more than a decade [46].

Human pressures have influenced the biota of the Gulf of Maine for thousands of years. Native Americans had caused declines in local cod stocks and changes in the coastal food web by 3500 BP [47]. In the 1700s, European colonists rapidly transformed the coastal environment, inducing widespread changes in abundance and diversity at all trophic levels [48]. By 1859 regional cod stocks on the Scotian Shelf were severely reduced [49], and by 1900 most large vertebrates in the southwestern Bay of Fundy were overexploited [48]. By the 1950s and '60s, most commercial fish stocks in the Gulf of Maine and Georges Bank were severely depleted [50]–[52], and many important stocks remain at low levels today [53]; in 2007, cod landings in the entire Gulf of Maine were only 5% to 6% of those in 1861 [54]. Fishing is still the greatest single anthropogenic stressor because of removals and trophic effects [3], [55]–[58] as well as impacts on benthic fauna and habitats [59]–[62]. There are many other existing and proposed uses of resources, including oil and gas development, sand and gravel mining, marine transportation, aquaculture, recreational fishing, and electrical generation from wind and tidal energy [63]. In the region, there is growing concern for biodiversity conservation and increasing recognition that marine management practices must be broadened to include the maintenance of habitats, biodiversity, and ecosystem functioning [64]–[67].

#### Management context

The Gulf of Maine is an international body of water shared by Canada and the United States, with the international border cutting across the northern Gulf of Maine and Georges Bank. Each nation has jurisdiction over its territorial waters. Although there are joint stock assessments and management plans for some transboundary fish species, such agreements are not legally binding and do not fully implement key principles of ecosystem approaches to management [68], [69].

Canada has a national Oceans Strategy that strives for sustainable development and recommends a precautionary approach when scientific information is lacking or incomplete [70]. The Department of Fisheries and Oceans is Canada's lead agency for managing the marine environment, which reduces the potential for interagency conflict and establishes a clear leadership role for implementing EBM [71]. In the United States, ocean governance is more fragmented, involving a number of federal, regional, state, and tribal authorities and jurisdictions, which can lead to overlapping and conflicting laws and regulations [72]–[74]. Some principles of EBM have been incorporated incrementally into U.S. management decision-making [14], and conservation of biodiversity is among the stated goals of many management agencies and laws. However, a more comprehensive integrated policy is needed for EBM to be fully operational and effective at a national scale [75].

### Research program motivations, design, and relative emphasis

The majority of field programs within the international Census of Marine Life were focused on the discovery of new species in underexplored areas of the world ocean [76]. The role of the GoMA study was to serve as the pilot project for a “whole ecosystem” approach to studying biodiversity at a regional scale [34] (Table 2). GoMA leaders perceived that a regional program of biodiversity research would do best over a long period of time if it promoted not only fundamental questions of composition, structure, and function, but also the application of biodiversity knowledge to sustainable management of a regional marine ecosystem. This linkage, it was argued, would help (i) build and maintain the funding support that would be needed to do the work; (ii) guide scientific program development (e.g., questions, scope, and dissemination of results); and (iii) ensure the use and impact of findings. GoMA started in 1999 with a series of workshops to assess what was known about the region's biodiversity [76] and to identify existing regional research and monitoring programs that would be key sources of information. These early workshops concluded that although there was a large body of knowledge about the region, there had not yet been any coordinated effort to summarize the Gulf's biodiversity in an accessible format [77] or to explore the connection between biodiversity and ecosystem function.

**Table 2:** Comparison of four regional marine biodiversity programs.

Category	Component	Subcomponent	GoMA	B-HMAP	GBRSB	GoMx
General	Primary drivers of program		Biodiversity, Ecosystem pilot study, Census project	Temporal change, Anthropogenic pressures, Census project	Fisheries sustainability assessment, Zoning assessment, Census affiliate	Update biotic inventory (book and online), Census affiliate
	Years	Lobbying	N/A	1999–2000	1996–2000	2002
		Planning/fund-raising	1999–2002	2000–2001	1999–2002	2003
		Implementation	2003–2010	2000–2010	2003–2007	2004–2010
Resources	# of countries/groups/agencies		2 countries, 5 universities, 3 NGOs, 2 national agencies, 2 state agencies	7 countries, 8 universities, 2 national fisheries laboratories, 1 museum, 1 government research institute	1 country, 5 research providers, 3 mgt agencies, 1 fishing industry association	15 countries, 140 authors, 80 institutions, 1 lead institution
	# of principal investigators		5	3	4	4
	# of people involved		>200	26	∼60	140
	Ship time (days)		165 (contributed)	5	305	N/A
	Estimated costs ($US)	Direct	6.3 M	170,000	∼1.7 M	0.3 M
		In-kind	3.1 M	470,000	∼5.8 M	1.5 M
	Sources of funding	Private	51%	26%	0%	80% (contributed)
		Government	49%	74%	100%	20%
Methods	Literature surveys & data collations		Y	Y	Y	Y
	Biological surveys		Y	N	Y	N
	Laboratory experiments		Y	N	N	N
	Acoustic surveys		Y (contributed)	N	Y	N
	Modeling		Y	Y	Y	N
	Paleoecological		N	Y	N	N
Results	# of new species documented		10 to date (>30 specimens awaiting further analysis)	0	Uncertain, possibly 1,000s	0
	Online species database		Y	Y	Y	Y
	Maps produced	Species distributions	Y	N	Y	Y
		Species ranges	N	N	Y	Y
		Habitat types	Y	N	Y	Y
		Bioregions	N	N	Y	N
	Environmental Impact Statements		N	N	Y	N
	Ecosystem Status Reports		N	Advice provided to European Environ. Agency Report on Marine Ecosystem Indicators	Input to GBR Outlook Report^2^	N
Management	Contributions		Developed framework for representing biodiversity knowledge within EBM^1^	ICES Baltic fisheries assessment WG; ICES workshops on a) historical data on fisheries and fish, and b) integration of environmental information into fisheries mgt strategies& advice	Trawl sustainability assessment; Zoning plan assessment	Track spread of invasive fish; Book and database used as references after an oil spill

1 [26].

2 [120].

(Program abbreviations as per Figure 2.) (Y = Yes, N = No).

#### Program design

The GoMA program began formally in 2003 with the following objectives:

- Synthesize current knowledge of biodiversity, including patterns of distribution, drivers of biodiversity patterns and change, and how biodiversity affects function of the Gulf of Maine ecosystem.
- Assess the extent of unknown biodiversity.
- Lead and support development of information systems to increase access to data.
- Support selected field projects and emerging research technologies.
- Work with the scientific community and individuals from federal agencies in the United States and Canada to help develop a framework for incorporating biodiversity information into EBM.
- Work with educators to introduce or strengthen biodiversity content in formal education settings.
- Educate the public on the role and importance of marine biodiversity.
- Recommend future research and monitoring.

To bring together existing information, GoMA supported development of a regional register of marine species as a tool to summarize the named marine species in the Gulf of Maine area. To bring forward spatial information, data and mapping applications were developed to serve regional biological and oceanographic data from multiple sources (Figure 3B). GoMA also worked with other organizations to develop a collaborative approach to data management and access. GoMA involved regional researchers by sponsoring, or cosponsoring, several small field studies and data assembly/analysis projects that explored new topics or areas, or enabled comparisons of biodiversity over time. Several U.S. projects that were planned and funded outside of GoMA became contributing partners to help advance the cause of coordinated and collaborative programs looking at biodiversity (e.g., two deepwater expeditions, a seamount study, a benthic observatory program, and an acoustic sampling development project). New proposals were initiated. Canada's Department of Fisheries and Oceans, in collaboration with several Canadian universities, conducted coastal fieldwork and four “Discovery Corridor” research cruises as contributions to the Census. GoMA also convened six binational expert groups that were organized around a combination of trophic and community types and habitats (coastal margins; zooplankton and pelagic nekton; benthos and demersal nekton; slope and seamount environments; microbial communities; and upper trophic level predators) to summarize what was known and unknown about biodiversity and its ecological role in the region and to recommend future research directions.

To ensure development of a realistic and useful model of how biodiversity knowledge could be used in public policy and management, GoMA worked with other groups and projects that emphasized stakeholder involvement, planning, and implementation. These included U.S. and Canadian fisheries agencies, which are working on implementing ecosystem approaches to fisheries management [67], [78] or integrated management approaches [71], [79], as well as academic, industry, nonfederal management, and conservation groups. From its inception, GoMA engaged science and policy advisers. GoMA also supported development of a collaborative and interdisciplinary network of educators and scientists working toward ocean literacy in the region, ensuring a prominent focus on marine biodiversity in education and in the public arena.

#### Relative focus on biodiversity elements

GoMA focused primarily on compositional and structural elements of biodiversity and less on functional elements (Table 3). Examples of compositional work include assembling a register of known species, investigating change over time at several well-studied sites, discovering new species, and exploring genetic patterns in populations. Examples of studies of structural elements include characterizing spatial patterns of diversity, analyzing diversity or compositional changes by geophysical mapping units, and examining genetic structure of invasive species to determine patterns of invasion. The influence of physical and environmental processes on species composition and structure was explored through statistical analyses; otherwise there was little emphasis, partially due to funding constraints, on how functional elements influence composition and structure.

**Table 3:** Program focus at a glance.

Principal Elements	Nested Elements	GoMA	B-HMAP	GBRSB	GoMx
Composition	Ecoregions	+ +	+ +	+ +	+ +
	Communities and ecosystems	+ +	+^1^	+ +	+
	Species populations	+ +	+ +^2^	+ +	+ +
	Genes	+	0	0	0
Structure	Ecoregional heterogeneity and complexity	+ +	+	+ +	+
	Habitat structure	+ +	+ +	+ +	+ +
	Population structure	+	+	+ +	+
	Genetic structure	+	0	0	0
Function	Ecological processes and disturbances	+	+ +	+ +	0
	Interspecific interactions, ecosystem processes	0	+ +	0	0
	Demographic processes	+	+	+	0
	Genetic processes	0	0	0	0

1 Mainly fish communities.

2 Mainly fish populations.

Relative focus of the four research programs on each of the elements of biodiversity outlined in Figure 1. Approximate proportion of effort allocated *within* each program is represented (+ +, strong focus of program; +, lesser focus; 0, not studied). No attempt has been made to scale effort across programs. (Program abbreviations as per Figure 2.) (Biodiversity classification approach developed by Noss [20] and by Cogan and Noji [21].)

### Research products and contributions to EBM

When the GoMA program started, no one knew how many named species there were in the Gulf of Maine area. The known species composition is now compiled in a Gulf of Maine Register of Marine Species (GoMRMS), an evolving, authoritative directory of GoMA species from microbes to whales, with references and electronic links to other sources of taxonomic and ecological information [26]. The register consists of both vetted and provisional entries and now includes over 5,600 species names.

About half of the species in GoMRMS have location information. GoMA worked with U.S. federal agencies and others to make spatially referenced biological data available through the Ocean Biogeographic Information System (OBIS) [80], facilitating the entry of more than 700,000 records for the Gulf of Maine area. Canada had already done this for shelf-scale and long-term demersal fish and invertebrate research trawl surveys, and OBIS now contains more than a million species/location records for the Gulf of Maine area. GoMA led the formation of the Gulf of Maine Ocean Data Partnership in 2004 with the goal of providing open access to regional biological, chemical, and geophysical data using recognized geospatial standards. In 2010, the partnership expanded southward to fit with the new organizational scale for ocean observing and regional ocean management in the United States (see Northeast Coastal and Ocean Data Partnership: http://necodp.org). The 25 partner institutions include Canada's Department of Fisheries and Oceans to bridge the international border that runs through the Gulf of Maine and Georges Bank.

Three expert groups have completed syntheses for the Gulf of Maine area, and the others are nearing completion. Li et al. [81] provided the first assessment of planktonic microbial (prokaryotic and single-celled eukaryotic) diversity and abundance and suggested that bacterial diversity information might be used as an indicator of spatial pattern and temporal change in ecosystem processes. Johnson et al. [82] synthesized data on species diversity of zooplankton and pelagic nekton, describing seasonal, regional and cross-shelf diversity patterns, and discussing possible trophic effects of climate-driven change on size and species composition of zooplankton. The authors suggested approaches for monitoring plankton and anticipating changes in the system, and listed several nektonic species (gelatinous zooplankton, euphausiids, mesopelagic fish, mysid shrimp, and squid) that require better assessments because of their important trophic roles. Kelly et al. [83] assembled the first comprehensive description of the biodiversity of the deep-sea continental margin of GoMA, including Bear Seamount. More than 1,670 species were documented for this subregion, which was more than expected; and analyses of species richness estimates suggest there are at least that many yet to be discovered [83].

A novel statistical analysis developed by Ellis et al. [84] was used to examine the relationship between benthic diversity and habitats on the continental shelf. The three largest fish and benthic invertebrate databases from the United States and Canada were used to explore relationships of fish and invertebrate biodiversity to 23 environmental variables such as depth, substrate type, bottom stress, temperature, and salinity. The physical variables predicted an average of 35% of the overall variation in biological patterns (abundance) of the 210 species encountered frequently enough for this analysis (out of 612 species in the databases, [85]). While this percentage of explained variance is fairly typical of marine ecological studies, this result indicates the limits of resolution when looking at the entire study area through these large historical surveys. By serving as a background, the results help guide the focus and interpretation of future studies.

#### Contributions to EBM

The contributions of GoMA to EBM include specific data products as well as new clarity about the relative breadth and depth of biodiversity knowledge about this regional ecosystem. The most tangible and immediately available results are the biodiversity databases: GoMRMS, regional contributions to OBIS, and the regional coastal and ocean data partnerships (GoMODP and NECODP). In conjunction with other developing databases, programs, and initiatives (Encyclopedia of Life, ocean observing, EBM), biological data for the Gulf of Maine are available for use by a larger group of users in more frequent and diverse ways than in the past. The expert group papers summarize highly specialized knowledge in a form accessible to nonspecialists and recommend broad (e.g., regional, trophic-level) research priorities applicable to EBM. The unique contributions of GoMA have been the inclusion of all taxonomic levels and all biodiversity, including the “unknown” as well as the known; a strong emphasis on data access and information systems; and an integrative spatial approach to the entire ecosystem.

In GoMA's early years, EBM remained a poorly defined and poorly constrained objective in the view of many (perhaps most) of the scientific community, and initially it was difficult to engage regional scientists in EBM-oriented discussions and research. In the years since, EBM has gained currency as a complex but necessary undertaking in which the conservation of biodiversity must play an essential role. GoMA contributed with other individuals and groups to achieve this general level of acceptance. Although a framework for connecting biodiversity science to environmental decision-making remains in early stages of construction, the concept of “ecosystem services” has been emerging as a possible bridge between the two disciplines [1], [27], [28]. Thus, it would be useful to develop one or two demonstration projects that focus on end-to-end analysis and modeling in comparatively small ocean spaces to describe quantitatively the links between biodiversity, ecological processes, ecosystem services, and management. This was proposed as a strategy in the early years of GoMA, and it remains an important approach. EBM at a regional scale will ultimately rely on insights gained from multiple scales and resolutions of study [12], [26], [86].

## Baltic Sea History of Marine Animal Populations: Case Study

### Regional description

#### Environmental context

**Figure 4:**
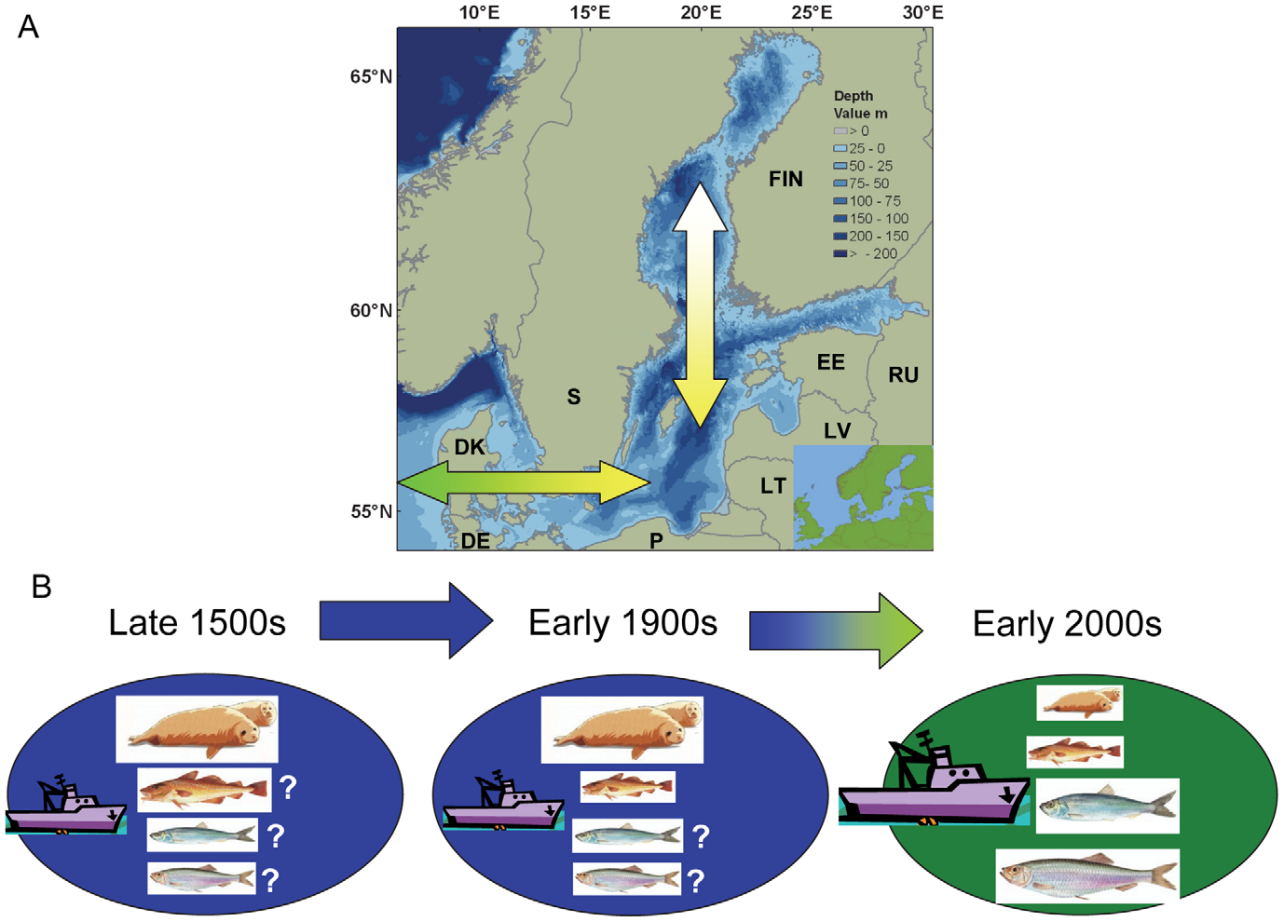
Map and representative data from the Baltic Sea History of Marine Animal Populations. (A) Map of Baltic Sea with adjacent countries (DK = Denmark, DE = Germany, FIN = Finland, EE = Estonia, LT = Lithuania, LV = Latvia, S = Sweden, P = Poland, RU = Russia). The green-yellow arrows represent the decrease in salinity from ca. 32 PSU to 2–5 PSU from the North Sea into the northern Baltic Sea. Map at lower right shows location of Baltic Sea on the European continent. (B) Schematic of changes in Baltic Sea ecosystems during the last 400–500 years. Sizes of animals (top to bottom: seals, cod, herring, and sprat) are proportional to their functional role and abundance in the system, and blue to green transitional arrows and oval backgrounds represent the change from an oligotrophic to eutrophic system during the twentieth century. Fishing intensity was substantially higher in the 2000s than in earlier centuries. Seals were much more abundant during and before the early 1900s; the biomass of cod was probably quite high in the late 1500s, but the biomass of herring and sprat is unknown before the mid-1970s (details in [96], [98], [105], [107]).

Situated at the border of the Atlantic and Euro-Asiatic climate systems, the Baltic Sea is one of the largest brackish water areas in the world. It is a semienclosed, epicontinental, nontidal and geologically young sea. The Baltic Sea is very shallow: about one-third of it is shallower than 25 m, while the average depth is approximately 60 m. The Baltic Sea is a heterogeneous system, encompassing 3 macroregions (the Transition Area, Baltic Proper, and Large Gulfs) comprised of 10 regions, some of which accommodate up to 4 subregions [87]. It is characterized by a southwest-northeast salinity gradient created by freshwater inflows mainly in the north, and saltwater intrusions in the southwest (Figure 4A). Another important gradient is the north-south temperature gradient. The northern Baltic Sea is almost entirely covered by ice in winter, whereas ice has been mostly absent in the southwest.

Biodiversity studies have a relatively long history in the region. Continuous datasets covering the entire Baltic Sea since the mid-1950s indicate that several groups of organisms are generally well studied, such as phytoplankton, mesozooplankton, macrozoobenthos, and fish. Currently, monitoring and study of these organisms is relatively well coordinated, and a unified methodology is applied across the whole sea (e.g., [88]). In contrast, information on several other organism groups, especially smaller organisms, is either relatively incomplete (for microzooplankton, meiobenthos, and disease-vectors and parasites) or extremely sparse (for heterotrophic bacteria). The most recent estimate suggests that today the Baltic Sea hosts over 6,065 named species [89]. Biota consists of natural immigrants of different origins: marine species, freshwater organisms, and glacial relicts.

Major stressors on biodiversity are harvesting of living resources, eutrophication, pollution, and more recently, invasion of alien species [89], [90]. Four fish species are internationally assessed and managed: cod, herring, sprat, and salmon. Currently, the two Baltic cod stocks and the Main Basin herring stock are depressed, while sprat and the gulf herring are relatively abundant. Baltic salmon stocks started to decline in the mid-nineteenth century. Several natural stocks have disappeared since then and catches are currently at low levels. Another major change in the biota of the Baltic Sea during the past century has been a drastic decrease in the abundance of marine mammals (Figure 4B). Initially this decline was mostly due to hunting, but since the 1970s it has resulted from toxic pollution. However, seal abundances have been recovering in recent years (e.g., [89] and references therein).

#### Management context

The Baltic Sea is bordered by nine countries: Denmark, Estonia, Finland, Germany, Latvia, Lithuania, Poland, Russian Federation, and Sweden (Figure 4A). The Baltic catchment area includes four additional countries [91]. Management of the Baltic Sea and its watershed is covered by numerous international conventions, directives, and policies focused on habitat, fisheries, specific groups of biota, and overall biodiversity (Text S1, see also [92]). Scientific advice for marine management is provided by the International Council for the Exploration of the Sea (ICES), which has recently undergone structural reform to provide EBM-type advice. The Helsinki Commission (HELCOM), the main forum for European environmental cooperation, has promoted the ecosystem approach since 2003 [93]. HELCOM's recently adopted comprehensive and rather ambitious Baltic Sea Action Plan relies on the ecosystem approach and addresses the region's most problematic issues, including eutrophication, hazardous substances, and maritime activities [92], [94]. The Plan specifically includes conservation of biodiversity as one of its main goals and provides concrete actions toward achieving defined ecological objectives [94].

### Research program motivations, design, and relative emphasis

In recent decades, there has been increasing recognition of the need for a historical perspective to understand human impacts on marine ecosystems and to restore, conserve, and manage marine animal populations. In response to this need, the Census funded a global multidisciplinary initiative called History of Marine Animal Populations (HMAP). The overall goals of HMAP were to improve understanding of long-term changes in the diversity, distribution, and abundance of marine organisms, the ecological impact of large-scale harvesting, and the role of marine resources in historical development of human societies [95]–[97].

The Baltic Sea was chosen as one of HMAP's case studies because its ecology has been relatively well studied over the past 50 years and some specific research questions could be formulated based on existing knowledge of ecological processes. Complementary historical data on fish and fisheries or paleo-ecological records that would help address these questions were also considered likely to exist. Some of the research questions that motivated historical studies of the Baltic Sea were related to the large-scale human-induced changes that occurred in the ecosystem during the twentieth century, namely, eutrophication, drastic reductions in marine mammal populations, and increased fishing pressure. Additionally, climate-driven hydrographic conditions (including salinity and oxygen concentrations), which strongly affect species distributions and biodiversity, varied widely in the last century [98].

The Baltic Sea History of Marine Animal Populations (B-HMAP) project that developed since 2000 (Table 2) is the first coordinated attempt to recover and make use of historical information on fish and fisheries on a Baltic-wide scale to improve understanding of the functioning of the Baltic Sea ecosystems and variations in biodiversity over time. These biological data and information potentially can be interpreted relative to multi-decadal, multi-century, and paleo-oceanographic hydrographic and climatic data for the Baltic Sea and northern European regions [99], [100].

#### Program design

The B-HMAP program studied regional marine biodiversity through temporal changes in some key species and the functioning of the ecosystem itself. This approach was chosen because of the relatively long documentation of various human impacts on certain species (seals, fish) and the overall ecosystem (eutrophication, pollution) (e.g., [98]). The research activities generally followed a three-step plan [29], [95] with the following goals:

- Identify ecological hypotheses related to long-term variations in abundance and catches of fish and marine mammals.
- Develop national overviews of the available materials and sources for all Baltic countries and identify which materials could potentially be the most useful and should be investigated in detail.
- Develop or modify hypotheses based on the established knowledge of the archival deposits, and undertake selected studies of the historical sources.

A major objective for B-HMAP was to create a dialogue among historians, archaeologists, paleo-ecologists, and fisheries and marine mammal ecologists. A second objective was to develop the knowledge of long-term processes affecting especially fish biodiversity and dynamics of key species and then apply this knowledge to the development of new baselines and conservation strategies for overexploited or suppressed living resources, and more generally to the management of the Baltic Sea ecosystem. The third objective was to disseminate the results to both the natural and social science communities.

The main sources of information that were considered potentially useful included quantitative sources such as annual tax accounts, customs rolls, household accounts, commercial catches, and scientific materials; qualitative sources, including fishing commissions' records, reports from government officers, public grants records, private sources and topographical literature; and archaeological documentation, which consists mainly of subfossil fish bones and evidence of fluctuations in fish productivity.

Quantitative approaches, including standard stock assessment models were applied to extend the knowledge of stock dynamics of the eastern Baltic cod in the twentieth century. For the other time periods and species, new information provided by B-HMAP was mainly related to catches and developments in fisheries, which in some situations are able to indicate qualitative developments in the fish stocks.

#### Relative focus on biodiversity elements

B-HMAP considered all three elements of biodiversity (Table 3). B-HMAP focused primarily on structural biodiversity within commercial fish stocks and the related ecological processes. The project considered the structure of habitats in the study area within regional ecosystem units and subregions [87]. Compositionally, the project covered a broad range of species/populations of different origins (marine, freshwater, migratory and glacial relict species), with different life history traits and environmental preferences in as many subsystems as possible. However, the project soon revealed the limitations in the availability of historical data, and consequently, the main focus became narrowed to only a few major marine species for which the most extensive data could be recovered with the available resources. These species were cod (essentially the eastern Baltic cod population) and herring, which have several distinct populations in the Baltic Sea. Fish population data were interpreted in the context of functional diversity elements such as human-induced and natural forcings (fishing, eutrophication, hydrography, predators), based on the most contemporary process-oriented knowledge (e.g., [101]).

### Research products and contributions to EBM

By establishing an international network of scientists and historians, important archival sources were identified and effectively mined for data. As a result, B-HMAP has located and made accessible a wealth of new materials for academic research by future natural and social scientists interested in fisheries and marine environmental history research [102].

B-HMAP quantified the taxonomic composition and magnitude of several fisheries in many of the countries around the Baltic Sea [96]. The contributions of different species to national catches varied among countries and over time, partly due to natural hydrographic gradients and temporal changes in abundance and in fishing technology. Most species exploited in the past are still exploited at present, but in lesser quantities in some cases. This applies particularly to the sturgeon and eel populations; the sturgeon is extinct in the Baltic zSea, and other studies have shown that eel biomass is severely reduced [103].

Some of the most important ecological results were related to the long-term (i.e., multi-decadal and multi-century scale) dynamics of individual key species. The project developed new time series extending into the early twentieth century [104], [105], when the Baltic ecosystem was in a different configuration than in the late twentieth century. The project also quantified the roles of climate, eutrophication, and exploitation in the development of the cod population. The project showed how the intensity of these forcings differed over time and also demonstrated how combinations of different forcings can have synergistic effects and consequently dramatic impacts on population dynamics [106]. In addition, the project was able to recover cod catch data for the late 1500s and early 1600s [107], which can potentially provide new estimates of cod biomass before intensive fishing, eutrophication, and marine mammal reduction (Figure 4B).

The long temporal perspective considered in the Baltic studies has increased understanding of how climate variability and change influence fish populations. Climate variability has been shown to have important impacts on all three of the most important commercial species (cod, herring, sprat), and temperatures in the Baltic in the early 2000s were warmer than at any time since the 1880s [96], [108]. Archaeological studies have shown that cod were abundant near Bornholm during the Atlantic Warm Period (ca. 7000–3900 B.C.), when temperatures resembled those likely to be typical in the late twenty-first century [109]. Although the warmer temperature might have been expected to reduce cod reproductive success and promote a cod-egg predator (sprat), the beneficial effects of higher salinity on egg and larval survival were probably dominant [109]. Such studies may help predict future impacts under projected hydroclimatic change. For instance, some preliminary model projections suggest the Baltic Sea may experience both higher temperatures and lower salinities [110]; this could lead to loss of marine fish species as fish communities become less diverse and are characterized by species tolerant of lower salinity [111].

#### Contributions to EBM

Results of B-HMAP are contributing to the establishment of new ecosystem and fishery management strategies for the Baltic Sea. These new strategies will need to accommodate multiple human impacts on the population and ecosystem as part of the efforts of HELCOM, ICES, and the European Union (EU) to move toward EBM in this region [112]. The results achieved here illustrate how these drivers have interacted in the past, the scales and magnitudes of variability, and possible baselines of abundance for recovery purposes.

For example, the new extended time series of biomass and recruitment [104], [105] indicate that high cod biomass was associated with a combination of reduced exploitation and good hydrographic conditions for reproduction, rather than reduced seal predation or nutrient concentrations [106]. Cod could become more abundant again under conditions where exploitation is kept at low to moderate levels, hydrographic conditions ensure sufficient reproductive success, and a moderate eutrophication level is achieved again [106]. Further, the historical ecological investigations illustrate how multiple forcings (e.g., fishing, hydrographic conditions) acting in the same or opposite direction can erode or promote resilience of exploited populations to collapse, suggesting that such considerations be taken into account when developing management policies [113]. Thus, B-HMAP results indicate that sustainable management of the Baltic Sea cod population will be most successful if it includes actions that reduce the risk of simultaneous negative impacts on cod productivity and increase the likelihood of multiple positive impacts on stock development [113]. There is recent evidence to support these suggestions: in the mid-2000s management measures to reduce Baltic cod exploitation levels coincided with good hydrographic conditions, resulting in nearly a fourfold increase in cod spawner biomass over four years [113], [114].

The new knowledge about interactions and dynamics of various forcings on fish populations and ecosystem structure will also be useful for comparative studies with other systems where cod and marine mammal populations still, or historically used to, interact [115] or where eutrophication and species invasions are, or will be, important drivers of fish population dynamics [116]–[118]. In addition, this new knowledge is applicable for development of integrated indicators of ecosystem status and health [119].

## Great Barrier Reef Seabed Biodiversity Project: Case Study

### Regional description

#### Environmental context

The Great Barrier Reef (GBR), the world's largest coral reef system, consists of almost 3,000 reefs spread along 2,300 km of shallow (≤100 m) continental shelf between the northeast Australian coast and the Coral Sea [120]. Living reefs comprise only 5% of the region by area and are found mostly on the outer half of the continental shelf, rising from depths of 30–70 m, or near the coast in depths of less than 20 m; the stretch of open water between is known as the GBR Lagoon. The shelf has alternated between terrestrial and marine environments over geological time due to changing sea levels, and present-day seabed habitats and biodiversity reflect this history while also being influenced by contemporary processes.

**Figure 5:**
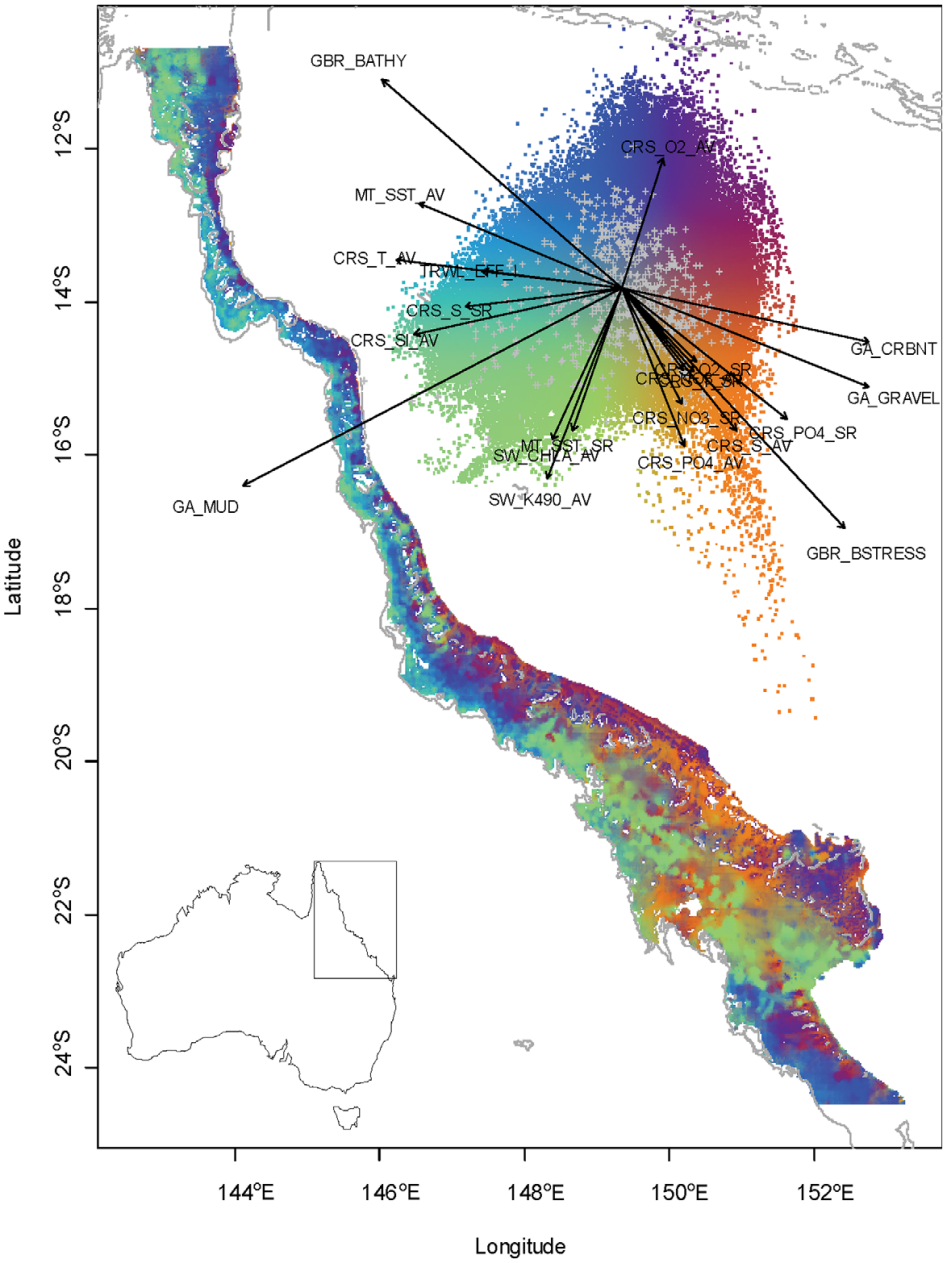
Map and representative data from the Great Barrier Reef Seabed Biodiversity Project. Biophysical map of the Great Barrier Reef continental shelf. Inset at top right: color key showing predicted patterns of biodiversity composition in 172,000 seabed 0.01° grid cells as represented by the first two dimensions of Canonical Correlation Analysis [173] of a data-matrix (850 species by 1,189 sites) constrained by 21 physical variables [140]. The labelled arrows indicate direction of major physical influences including sediment composition, current stress, bathymetry, ocean color, and bottom-water chemistry. The characterization is mapped to geographic space showing the location of these physically influenced seabed assemblages and is indicative of the stratification in the sampling design of the study.

In the north, the shelf is generally shallow and as narrow as 20 km, with ribbon-reefs at the shelf edge forming a nearly continuous barrier. In the south, the shelf broadens to as much as 260 km and generally is deeper; the outer shelf is dominated by two lines of dense reefs separated by narrow channels (see [121] for detailed discussion; see Figure 5 for physically influenced patterns of seabed biodiversity). The southern GBR is tidally dominated, with tidal range up to 10 m in places, causing extreme tidal currents. Such currents scour away sediments, progressively depositing them in less energetic areas. The far northern GBR also has very strong tidal currents, because of the out-of-phase tides of the Coral and Timor seas [122].

Coastal processes, in particular terrigenous sediment export from rivers, influence the inner shelf. During floods, turbid plumes of dissolved and suspended materials enter the GBR Lagoon, where winds drive them northward along the coast [123] depositing a thick wedge of muddy sediments up to 15–20 km wide. Beyond the coastal zone, tropical storms (cyclones) are the major force redistributing deeper shelf sediments north and inshore, leaving only a thin veneer of coarse biogenic carbonate debris [124]. Oceanic processes influence the outer shelf, forced by the westerly South Equatorial Current, which bifurcates at the GBR continental margin at about 15°S, producing a northward boundary flow and the southward East Australia Current. The outer ribbon-reef barrier in the north restricts on-shelf exchange of oceanic water, as do the two lines of dense reef development in the south. In the central GBR, the more open reef matrix and deeper outer shelf allow episodic upwelling of oceanic water onto and across the shelf [125].

The GBR is characterized by high biological diversity and lies on the southern fringe of the Indo-West Pacific biodiversity hot spot known as the “Coral Triangle.” It supports many thousands of species of all major groups of marine animals and plants [120]. The GBR shelf encompasses the Northeast Australian Province [126] within the central and southern two-thirds of the region. It is bounded to the south, in the vicinity of Fraser Island (∼25°S), by the Central Eastern Subtropical Transition. The northern GBR and adjacent Torres Strait (∼10°S), which was emergent during glacial periods isolating GBR biota from northern Australia, make up the Northeast Tropical Transition.

European settlement of the GBR catchment began more than 150 years ago, though rapid economic development occurred later, in the 1960s. The GBR was then considered an untapped resource to be exploited: exploration for oil commenced and mining was proposed; fishing expanded as new fisheries opened and unregulated foreign fishing increased on offshore reefs and waters; and tourism began to boom [127]. Development of grazing, farming, industry, and urban areas on the coast and in catchments increased soil erosion and export of sediments to inshore ecosystems, raising concerns related to effects of increased turbidity and sedimentation on coastal coral reefs [128], and runoff carrying pollutants, including fertilizers and pesticides [129]. Outbreaks of the crown-of-thorns starfish (*Acanthaster planci*) became increasingly frequent, and anthropogenic causes were implicated [127]. Rising public concern about these unrestrained development issues, particularly oil drilling, ultimately led to the declaration of the GBR Marine Park in 1975 and the creation of a new management agency, the Great Barrier Reef Marine Park Authority (GBRMPA). While conservation and protection of the new park were paramount, a broader multiple-use philosophy guided regulation of reasonable uses of the GBR to minimize their effects and enable some areas to be reserved for public appreciation and others to be preserved undisturbed [130]. This approach represented early progress toward EBM.

#### Management context

The GBR is highly valued nationally and globally, and after being protected in 1975 as a marine park it was declared in 1981 as a World Heritage Area representing an outstanding example of Earth's biological and geological evolution and processes [131]. This status brings international obligations to ensure the protection and conservation of the area to preserve it for future generations. While there are no longer threats from oil drilling and mining, many of the other concerns remain and new concerns have arisen, including climate change and associated risks of coral bleaching, future ocean acidification, and increased cyclone intensity [132]. The challenges for management are to ensure that multiple human uses are sustainable, balancing the economic contributions from tourism, transport, and fishing with the benefits of recreational activities and the conservation of natural resources and values, in a context of external global changes [120], [131], [133]. Except for commercial fishing, numbers and types of uses of the region are increasing. Management responsibility lies primarily with the GBRMPA, a Commonwealth (national) government authority supported by explicit governance relationships with other Commonwealth and Queensland (state) government departments and the involvement of nongovernmental organizations and local community-based committees. The key management mechanisms involve spatial zoning and regulation of activities by permits.

Commercial and recreational fishing are the major extractive uses of the region's biological resources. The 10 major commercial fisheries are licensed limited entry, and their footprint has reduced over the last decade [120]. Trawling is the largest and most valuable fishery, harvesting about 7,000 t of prawns and scallops annually (Queensland Department of Employment, Economic Development and Innovation, unpublished). Trawling is also the most widespread activity on the seabed. Previous research in the GBR has shown that trawling can have direct impacts [134], particularly on biota that is easily removed or slow to recover [135]. In response, management closed untrawled areas and capped effort in 1999, then progressively reduced effort. Further, the entire Marine Park was rezoned in 2004, increasing zones protected from trawling from 25% to 56% of shelf area [136], [137]. The previous studies recommended that the regional distribution of vulnerable habitats and biota be mapped to assess the environmental sustainability of trawling and the efficacy of management regimes [138].

### Research program motivations, design, and relative emphasis

Assessing the environmental sustainability of the trawl fishery by mapping seabed habitats and biodiversity on the GBR shelf, which previously had been surveyed extensively only for seagrasses [139], was a major driver for the GBR Seabed Biodiversity Project (GBRSB) [140]. Primary goals were to provide information for biodiversity assessment and future planning needs and to support an ecologically based approach to management of the fishery [140]. The science drivers included basic research in biodiversity and biogeography, including new species discovery and evaluation of physical and biological surrogacy.

Specific objectives of the GBRSB were as follows:

- Produce comprehensive inventories and maps of the distribution and abundance of species, habitats and assemblages.
- Analyze biophysical relationships and assess the utility of environmental variables for spatial prediction of biological distributions.
- Provide attributes (e.g., biomass, species richness, rarity, uniqueness, condition, potential vulnerability) of biological assemblages.
- Develop quantitative indicators of exposure to and effects of trawling, and indicators of sustainability risk.
- Populate and run a dynamic model of the status of structural fauna and evaluate the ecological benefits of recent management strategies.
- Quantify the large-scale effects of trawling on bycatch species and benthos assemblages.

The combined skills and resources of several research providers and support from multiple funding agencies were necessary to meet the multidisciplinary objectives of the project (summarized in Table 2). By providing biomass distributions of seabed species, this study would add important new information to 15 years of previous research on the effects of trawling and recovery, enable the scaling up of local experimental results to increase understanding of the effects of trawling at regional scales, and allow evaluation of the efficacy of management strategies [134], [141], [142].

#### Program design

The design phase of GBRSB brought together large-scale datasets such as bathymetry, sediments, oceanographic model output, water chemistry, and ocean color (including their seasonal variability where appropriate) as well as available existing biological sample data; all were mapped onto a set of 0.01° grid cells. Relationships between the existing biological and physical data were analyzed to identify and rank physical variables important for structuring patterns in seabed biodiversity. The biological importance of variables and their inverse density distributions were used to stratify and weight the grid cells [143]. Sites for sampling were selected from these strata to provide representative coverage of the environment space in the region. Because of the high cost of surveying for detailed biodiversity distribution information, the available environmental data layers were used as surrogates for predicting biodiversity distributions to maximize the utility of samples collected. Evaluating the performance of biophysical prediction and its implementation in sampling design and analyses for seabed biodiversity mapping was central to the approach of GBRSB.

The operational phase of the project mapped seabed habitats and their biodiversity throughout the continental shelf in the GBR region by visiting nearly 1,400 sites, stratified along multiple influential environmental gradients (Figure 5). Multiple devices were deployed at each site, including towed video and digital cameras, baited remote underwater video stations, an epibenthic sled, and a research trawl to collect samples for detailed data about plant, invertebrate, and fish species on the seabed [140]. Challenges due to adverse weather, equipment downtime, and other difficult conditions at sea led to gaps in survey coverage, as did restrictions on extractive sampling in high protection zones. Limited storage capacity for samples on vessels, meant that subsampling was necessary though undesired. The sheer volume of samples required setting priorities among biotic groups for processing, leaving some more difficult groups unsorted. Ultimately, additional resources were obtained from partners to support extra voyages and staff, to address the most critical gaps in sampling and processing.

In a previous project, a dynamic spatial modeling tool had been developed to compare trawl management options within the Marine Park area [142]. The model simulated the impacts of trawling on sessile benthic fauna in terms of relative biomass removal, by implementing the removal and recovery rates measured in empirical studies, and the spatial and temporal behavior of the trawl fleet. The model was run again with the new species distribution maps from the GBRSB project to estimate the regional-scale impact of past trawling on susceptible sessile benthic species and evaluate the outcomes of management interventions since 1999.

#### Relative focus on biodiversity elements

The GBRSB focused primarily on compositional and structural elements at intermediate to large scales within the GBR ecoregion and focused less on functional elements (Table 3). Sampling was directed at compositional elements, specifically the identity and inventories of habitat types and species abundance at sites, including new species discovery. Structural elements included maps of species distributions showing patterns of assemblages at bioregional, landscape, and community spatial scales; characterization and distribution of the habitat structure of biophysical regions; species biomass distributions and their pattern of beta diversity associated with physical and environmental attributes. Functional elements were primarily related to regional-scale conservation and resource use issues, including risk assessment of the effects of trawling, and evaluation of the effectiveness of management interventions, including marine protected areas. These assessments incorporated demographic processes and life history information from related projects. The potential functional influence of physical and environmental processes on composition and structure was investigated through statistical analyses.

### Research products and contributions to EBM

The shelf seabed biodiversity of a high profile World Heritage Area and multiple-use marine park was largely “unknown,” but now — after only three years of intensive sampling — it is “known” in considerable detail. As a species and habitat inventory, the project recorded more than 5,300 marine organisms, many of which were new species or new records for Australia, all with cataloged museum voucher specimens that will serve taxonomic and genetic investigations into the future. Additional resources include video footage of more than 150 seabed habitat types and more than 300 fishes, sharks, rays, and sea snakes. The project generated a database of more than 140,000 records of species distribution and abundance on the seabed, along with digital maps of the distribution and abundance of about 850 seabed species.

A multidimensional complex of environmental variables was identified as influencing seabed species distributions, including sediment grain size and carbonate composition, benthic irradiance, current stress, bathymetry, bottom water physical attributes, nutrients, and turbidity (e.g., Figure 5). Predictive biophysical models were developed linking seabed species, their assemblages, and the physical environment. These developments are now aiding the prediction of seabed biodiversity patterns elsewhere in Australia and internationally.

Estimates of the likely extent of past effects of trawling on non-targeted benthos and bycatch over the entire shelf of the GBR region indicated that trawl effort had a significant effect on the biomass of only 6.5% of the 850 mapped species — a negative biomass change for some species and a positive biomass change for others. Estimates of exposure of species distributions to contemporary trawl effort distribution showed that about 70% of the 850 mapped species had low, or very low, exposure to trawl effort. At the other extreme, about 33 species had high, or very high, exposure to trawl effort. Three species exceeded a limit reference point analogous to maximum sustainable yield, and another 10 species were also considered as high risk even though they were below the sustainability reference point, because of uncertainty in parameters (see [140] for more details).

Model-based evaluations of the environmental performance of several recent management interventions showed that generalized depletion trends of sessile benthic fauna predicted until the late 1990s have all been arrested and reversed [140]. A buyback of fishing licenses in 2001 and subsequent removal of effort, through penalty provisions on effort-unit transfers and vessel replacements, made the biggest positive contributions, and the 2004 rezoning of the marine park produced additional gains for some species.

#### Contributions to EBM

Ecological risk and sustainability indicators and biological reference points for the trawl fishery were developed by GBRSB with management and industry involvement. These showed that the majority of nontargeted biota vulnerable to trawling have distributions that overlap little with trawl effort. Evaluations of recent management changes indicated that prior unsustainable trends of sessile benthic fauna have been arrested and reversed. Such information is contributing to statutory ecological assessments for the trawl fishery, necessary criteria for product export approvals, enhancement of regular fishery performance monitoring with respect to environmental sustainability, complementary ecological risk assessments, and revision of the *Fisheries (East Coast Trawl) Management Plan 1999* under the principles of ecosystem-based fishery management. The GBRSB outputs have been used again in 2010, along with 2009 data on the distribution and intensity of trawl effort, to recalculate the exposure and sustainability indicators for habitats, assemblages, and higher-risk species from the 2005 assessment. This update showed that further reductions and contractions in trawl effort have corresponded to reduced exposure and risk in all cases (Pitcher unpublished workshop presentation).

The results of GBRSB were used to assess the effectiveness of the 2004 rezoning [144] in meeting its stated objectives of conserving at least 20% of habitats and biodiversity within 40 benthic bioregions recognized by GBRMPA's Representative Areas Program [136]. While the Representative Areas Program had access to some of the same physical datasets used to stratify the sampling effort in GBRSB, direct data on seabed species and habitats were sparse. As such, the Representative Areas Program bioregionalization was an expectation based on physical surrogates, guided by delphic interpretation of available expert opinion. The quantitative post-hoc analysis made possible by the GBRSB showed that the re-zoning met or exceeded the target for biodiversity conservation [144]. In fact, the average increase in level of protection conferred to seabed assemblages was about 30%, closely matching the increase in area of seabed reserved in “no-trawling” zones. This outcome demonstrates that important environmental variables can be used as effective surrogates for conservation planning where knowledge of the biology is imperfect; this can be especially helpful in marine benthic systems, which are difficult and expensive to study and often have poorer historical coverage than their terrestrial or pelagic equivalents [145].

The knowledge arising from these assessments has led to greater assurance that seabed biodiversity is unlikely to be at significant ongoing risk from trawling and that management is helping to conserve the diversity of benthic habitats and biota in the Great Barrier Reef. This has, in turn, quelled the previously polarized debate over trawling in the region. The skills and experience of GBRSB were adopted into the Commonwealth Environment Research Facilities Program's Marine Biodiversity Hub, which has progressed to predictive mapping of seabed biodiversity patterns at the Australian national scale for regional planning and conservation [146]. Some of these analytical approaches have been applied in the Gulf of Maine and Gulf of Mexico regions [85]. GBRSB outputs have also contributed to ongoing marine park planning and reporting, including the GBR Outlook Report [120] and current reporting to UNESCO World Heritage Center.

## Gulf of Mexico Biodiversity Assessment: Case Study

### Regional description

#### Environmental context

**Figure 6:**
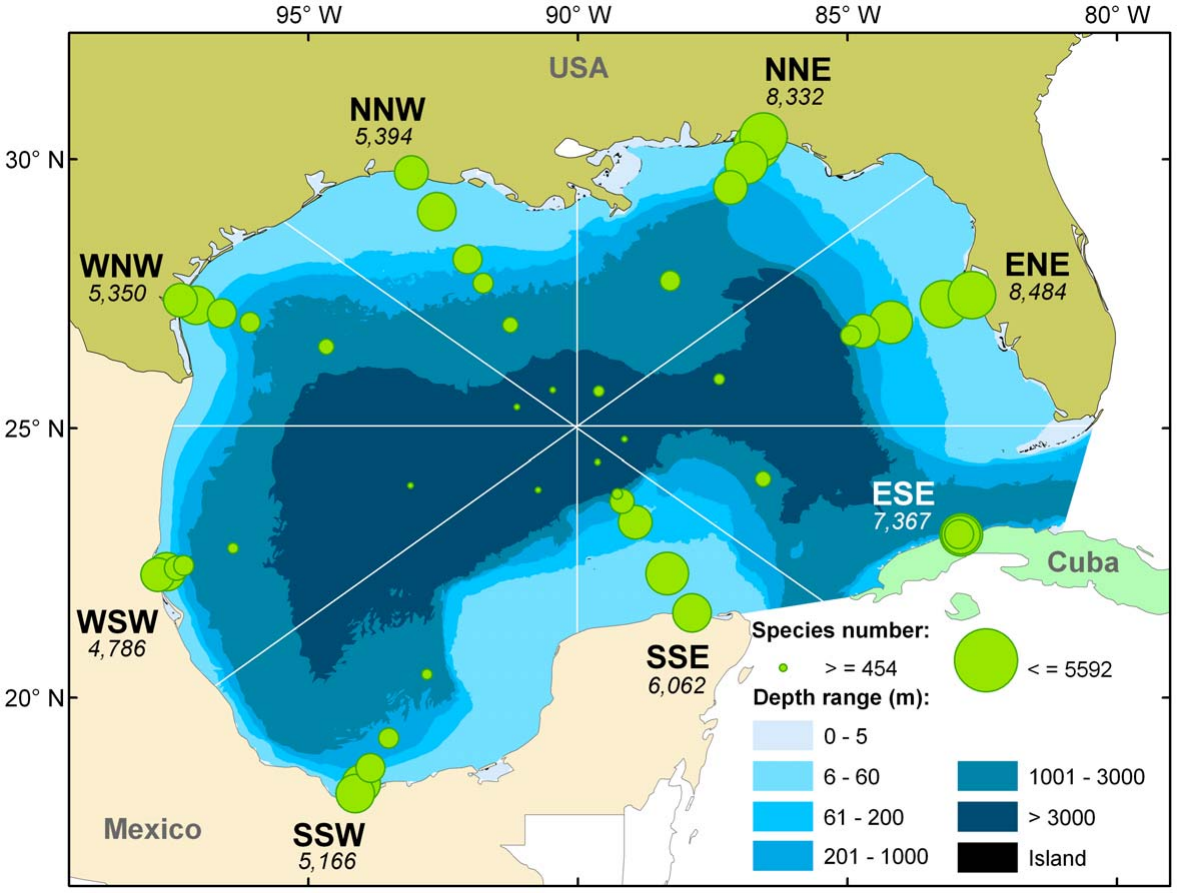
Map with representative data from the Gulf of Mexico Biodiversity Assessment. Spatial representation of marine biodiversity by sector and depth. The Gulf was divided into four quadrants of approximately equal areas, then further divided into eight sectors, each of which was divided into six depth ranges (polygons). The information on species ranges was converted into a searchable digital database, allowing for spatial examination of marine diversity. Numbers under each sector heading indicate the number of species recorded in that sector. The size of the circles is proportional to the number of species occurring in each polygon.

The Gulf of Mexico is surrounded by the United States, Mexico, and Cuba (Figure 6). It is the world's seventh largest peripheral sea, with a surface area of 1.51 million km^2^ [147] and a volume of 2.4 million km^3^ [148]. The Gulf has a central basin surrounded by a shallow rim; it is sometimes considered the third coast of North America, or America's Mediterranean Sea. Although considered a shallow sea because of its broad continental shelves (<200 m depth) that make up 32% of its area, it is on average quite deep (Table 1). Warm tropical water enters from the Caribbean Sea via the Yucatan Straits (Figure 6), where it forms the Loop Current. The Loop Current flows to the Atlantic through the Florida Straits and forms one of the world's strongest currents, the Gulf Stream. The Mississippi River drains 41% of the U.S. watershed and is the principal source of freshwater inflow into the Gulf [149]. More than 80% of the Gulf of Mexico is covered by silty to sandy sediments from the Mississippi, with smaller contributions by other rivers [150], yet the Gulf has diverse benthic habitats, including salt diapirs, pinnacles, benthic brine pools, mud volcanoes, drowned Pleistocene coral reefs, depositional remnants, and shell banks. In deeper waters, hydrocarbon seeps have resulted in unusual carbonate buildups, formation of biogenic carbonates, gas hydrate beds, and asphalt plains [151]. More than 4,000 oil and gas platforms and thousands of miles of pipeline occur throughout the Gulf, contributing additional artificial substrates.

The great habitat complexity is thought to support the region's high biodiversity (Table 1), which is due to both endemic and cosmopolitan species [152]. Biogeographically, the shallow waters of the north are warm temperate (Carolinian Province) and those in the south are tropical (Caribbean Province) [153], although the boundaries of these provinces are subject to debate [154]. Modern stressors on biodiversity include habitat loss, overfishing, and degradation of water quality [42]. Harmful algal blooms and hypoxia frequently drive mobile animals from certain areas, and increasing coastal development and recent intense hurricanes have been destroying or eliminating coastal habitats [42].

#### Management context

A number of international, federal, regional, and state jurisdictions oversee management of biotic resources in the Gulf of Mexico. The United States, Mexico, and Cuba manage fisheries and other resources within their 200-mile exclusive economic zones (EEZs), with minimal interactions between the United States and Mexico for oversight of migratory species, and little to no interaction with Cuba. One area known as the “Western Gap” lies outside the 200-mile EEZs of the United States and Mexico; this zone, slightly larger than the state of New Jersey, reportedly contains commercial quantities of oil and gas resources [155] and is a spawning area for bluefin tuna [156]. No regulatory oversight occurs in the Western Gap.

Within the U.S. EEZ of the Gulf of Mexico, the Southeast Region of the National Marine Fisheries Service is primarily responsible for sustainable fisheries management, habitat conservation, and protected resources management. The Southeast Region provides technical and administrative support for the Gulf of Mexico Fisheries Council, which prepares fisheries management plans for species in Gulf waters [157].

### Research program motivations, design, and relative emphasis

The first comprehensive biotic inventory of the Gulf of Mexico was compiled in 1954, when 55 scientists published *Gulf of Mexico: Its Origin, Waters and Marine Life* [158]. In this landmark volume, regionally known as Bulletin 89, 44 taxonomists produced the most reliable regional taxonomic synthesis; they listed 2,444 species in 30 phyla and domains. Many more species were known from the region at the time but either were not included in the volume or were treated only summarily, and the detail of coverage varied between taxa. For instance, no decapod crustaceans were listed, and only a few mollusks were included. The zoogeographic and bathymetric ranges of most species were not provided, and little standardization existed among the different chapters. Despite these shortcomings, Bulletin 89 was the standard reference for faunal studies of the Gulf. The volume also contained sections on the history of explorations, geology, meteorology, physical and chemical oceanography, and pollution. The biotic inventory comprised 387 pages of the 664-page volume.

One of the earliest goals of the recently endowed Harte Research Institute for Gulf of Mexico Studies (HRI) at Texas A&M University-Corpus Christi was to conduct a 50-year update of Bulletin 89, with a separate volume dedicated to the biotic inventory. This effort would support HRI's mission to promote the conservation of biodiversity and the sustainable use of resources in the Gulf of Mexico. In October 2003, HRI hosted a State of Knowledge Workshop to establish guidelines for an update of the Gulf's biotic inventory.

#### Program design

The Gulf of Mexico Biodiversity Assessment (GoMx), coordinated by HRI with partners in the United States, Mexico, and Cuba, assembled a multinational group of contributors to the biodiversity volume (Table 2). A detailed set of guidelines and formats was developed to provide authors a template to report their results. This template had to be modified slightly for a few taxonomic groups because some of the information did not apply to all groups (e.g., depth for marine avifauna, or host species names for nonparasitic taxa), but overall the final product was quite uniform. Authors were required to provide a short introduction to the taxonomic group of their expertise, a review of pertinent literature in the Gulf of Mexico, a comprehensive listing of all living species with well-documented records in the Gulf of Mexico, and an updated taxonomy. The template for tables of species checklists consisted of rigidly defined columns for the following information: taxon, including higher taxa, with the species name and authority and date; habitat-biology, depth in meters; overall geographic range; Gulf of Mexico range; and references or endnotes. In addition to searching published literature, each contributor to the biodiversity volume accessed specimen collections and records of many regional and nation museums, as well as personal collections, to verify the identification and range of species included in the volume.

The Gulf of Mexico was defined geographically as the marine habitats, coastal waters, and tidal wetlands in the Gulf proper and northwest Cuba. The limits of the Gulf were defined as the line between Cabo Catoche, Quintana Roo, Mexico, and Cabo de San Antonio, Cuba, and the line between Punta Hicacos, Cuba, and Key Largo, Florida (Figure 6). The Gulf was divided into four quadrants of approximately equal areas; the 90°W meridian divides the Gulf of Mexico into western and eastern halves, and the 25°N parallel divides the Gulf into northern and southern halves (Figure 6). This division was for practical reasons in reporting distribution of species in the Gulf, not for biogeographical boundaries. Each quadrant was further divided into two sectors, resulting in eight sectors that could be used to report species with detailed distribution within the Gulf. The taxonomic authors of the volume volunteered their time and many were not familiar with geographic information systems software, thus their early decision was to report species distributions only as geographic ranges (within quadrants) instead of reporting coordinates for each species.

#### Relative focus on biodiversity elements

GoMx focused primarily on species composition. However, the assessment included structural data in the form of species spatial distribution data (at large scales - octants of the Gulf) and also the bathymetric and habitat distributions of the species. Functional elements, such as ecological interactions and assemblages, were not treated but will be considered in the next phase of the project, which includes the creation of a digital database with a toolkit. Structural elements included standardized tables (for each taxon), which list the spatial distribution of species within the Gulf and worldwide, depth distribution, and habitat affinities. Functional elements could be derived from this data and applied to regional-scale conservation and resource issues, such as spatial risk assessments of the effects of exploration, extraction, and transport of minerals and hydrocarbons from different regions of the Gulf.

### Research products and contributions to EBM

The resulting biodiversity volume [159] took four years to complete and includes all known living species in the Gulf of Mexico, from bacteria to plants, and from invertebrates to vertebrates. The volume is arranged in 77 chapters, accompanied by an introduction and a chapter on population genetics. It resulted from the collaboration of 140 taxonomists from 80 institutions in 15 countries (Table 2), who compiled a comprehensive inventory of 15,419 species from 40 phyla and domains. A total of 1,263 species are potentially endemic to the Gulf, while 552 are believed nonindigenous to the region. The sixfold increase in the number of species from the original Bulletin 89 is the result of a more comprehensive taxonomic, geographic, and bathymetric coverage, new species descriptions, range extensions, reports of nonindigenous species, and many new expeditions and research efforts, including the exploration of deep water habitats with manned submersibles, remotely operated vehicles, and drop-camera systems.

The species checklist has been converted into a digital database, compatible with OBIS. The database used a simple but comprehensive biodiversity data model to integrate taxonomy, distribution, habitat, depth, and bibliographic references of all species. The resulting database constitutes an organized, searchable database management system named Biodiversity of the Gulf of Mexico Database (BioGoMx). The first online version of BioGoMx was published in January 2010 in collaboration with OBIS-USA. In March 2010, the database also became accessible through international OBIS. This biotic database constitutes a science-based guide for the scientific and conservation communities. Because the BioGoMx dataset is richer than the data served by OBIS, it will also be accessible via GulfBase (http://gulfbase.org), a portal developed by HRI for data on researchers, institutions, expeditions, and environmental conditions. The BioGoMx database provides additional information for all species reported in the biodiversity volume, including habitat, biology, and depth, which species are endangered, endemic, or nonindigenous, and pertinent references, synonyms, and other data that were provided as footnotes. The database allows for corrections and new data, such as new records, range extensions, and new species.

#### Contributions to EBM

The ultimate outcome of GoMx will contribute to better-informed decision-making by resource managers, researchers, and the public. The species inventory will become a standard reference and benchmark to help verify biotic studies and to assess status of species including range extensions, community assemblages, introduction of invasive species, or other anthropogenic or natural environmental change. Because habitats were included in the species checklist, aspects of changes in habitat by species could also be studied. The database can be mined for zoogeographic investigations of species and communities, biodiversity studies, planning environmental impact assessments, determining optimal locations for marine protected areas, and EBM. Researchers will be able to use the inventory to ask which region or depth might present the highest potential for biodiversity or habitat loss, or which region has the highest percentage of endemic species or nonindigenous species; similar questions could be posed for harvesting commercially important species. The species inventory will serve as an essential tool for comparative zoogeographic and biodiversity studies with other Large Marine Ecosystems.

One recent application has been to track the introduction and range expansion of an invasive species. The red lionfish (*Pterois volitans*) is a venomous species normally indigenous to the South Pacific Ocean and had not been reported from the Gulf at the time of publication of the volume. This rapidly spreading species has since been collected in numerous locations in the eastern and northern Gulf and has been added to the BioGoMx database of the 554 species listed in the volume as nonindigenous to the Gulf. Another high-profile application of the all-species inventory has been in response to the 2010 Deep Horizon oil well blowout in northern Gulf of Mexico. As researchers have been planning projects to determine the effects of the oil spill on the biotic communities of the region, the volume has been in high demand as a reference for the correct taxonomy, distribution, and depth range of species. In addition to standardizing the taxonomy of species and providing contact information of taxonomic experts, the inventory is being used to determine whether spatial and bathymetric distributions of species were altered by the disaster.

## Discussion

### Program design and achievements

Each program described above was designed to improve understanding at the ecosystem scale, but they were created independently with differing objectives, scopes of inquiry, and approaches. Three programs (GBRSB, GoMA, and GoMx) specifically pursued taxonomically broad biodiversity assessments and emphasized the importance of spatial patterns, albeit at varying degrees of spatial resolution (GBRSB>GoMA>GoMx). B-HMAP focused on a subset of commercial fish species and their primary prey and predators as conspicuous indicators of system change and used a historical approach to evaluate the relative roles of climate change and anthropogenic forcing. All four programs lasted 8–10 years, including several years to develop interdisciplinary networks among scientists, policy experts, managers, social scientists, or historians (Table 2). Lead time was also needed to create agreement on how to move ahead and to raise sufficient funding. GoMx and B-HMAP had to coordinate this process in numerous countries, which was easier for B-HMAP because mechanisms for European scientific cooperation already existed. The challenge for B-HMAP was to create support for a historical analysis whose feasibility had not yet been demonstrated. GoMA had a simpler international challenge because only two countries were involved; however, despite a history of scientific and management cooperation between the two countries, scientific funding mechanisms and cycles differ, and there is no binding international arrangement for how ocean space is to be managed. It thus took time to create a truly binational effort. Although GBRSB involved a single nation, the level of debate concerning objectives and approaches nevertheless was intense, probably because this program involved the greatest investment in new sampling and the closest ties between the forthcoming scientific results and management implications.

All four programs resulted in new or substantially revised data systems. For GBRSB and GoMA, those systems specifically included ties to geophysical and other environmental data in a broad “ocean observing” context. Three programs yielded substantially revised inventories of known species. GoMA and GoMx accomplished this through searches for, and assembly of, previous records, whereas GBRSB initiated a significant exploratory field project. GoMx, which was focused exclusively on species inventory and entrained 140 taxonomic experts, has published a validated list. In contrast, GoMA and GBRSB have many species names or identifications yet to be vetted, and many samples await examination. During this time, a new inventory of Baltic biodiversity was assembled [89], but separately from B-HMAP. In their own way, each of these programs established important resources of information. They also revealed the scope of work needed to describe their respective systems (even partially) in order to serve regional needs for sustainable management (EBM) and meet international goals for elucidation and conservation of biodiversity (e.g., [160]).

To date, there have been differing extents of delivery and use of scientific information to and by management. GBRSB was the most fully integrated in this respect, because the study was designed around specific management questions, and the design was the product of intense deliberation between managers, scientists, industry, and conservation interests. Information has already been used in fishery and conservation planning, including ecological risk analyses. Importantly, the scientific design of GBRSB also enables the use of data in other ecological applications directed at the seabed. There is a lesson contained in this history, because there was pressure initially to design a less expensive sampling program sufficient to address only some of the immediate management questions. There is now broad agreement that the more rigorous sampling program that was conducted was a good investment. The other three programs were not designed with specific management questions in mind, but rather sought to advance biodiversity knowledge as a public resource available for research and management. Among the four programs, GoMA worked most explicitly to define links between curiosity-driven science and issue-directed science for EBM applications [26], [34]. B-HMAP delivered data products that could be used in anticipating the future of fisheries based on climatic conditions, an important but limited view of system biodiversity. GoMx's list and description of species and their ranges is the most structured and comprehensive of the four projects and is a public resource available for many uses, but it is not as spatially explicit as the other projects.

Each of the projects has identified link points between the data and insights they have generated and EBM applications; however, to secure broader advances and tighter linkages, agreement on the approach and scope of what should be done next is required. While the need for information is immediate, there is an almost unlimited number of interesting questions, there is no consensus on how to approach the large number of centrally important questions, and the field work and analysis are expensive and time-consuming. One of the challenges is to define a framework for discussion within which the domains of curiosity-driven and issue-driven investigations can be visualized and inform each other. Although the immediate applications of curiosity-driven science may not be apparent to management, it is often this branch of science that makes new discoveries, insights, and innovations that become important to management.

### Relative program emphasis on composition, structure, and function

Biodiversity is an essential consideration in managing human activities in marine ecosystems, but this term encompasses a wide spectrum of biological attributes and ecological processes. Translating the general objective of “biodiversity conservation” into a series of recommended scientific investigations and management guidelines is difficult, and the use of compositional, structural, and functional elements provides a mechanism to highlight a suite of related topics and describe how they fit into the knowledge needed to implement EBM. No single program is likely to examine all three biodiversity elements of an ecosystem equally. Indeed, the utility of this deconstruction is to make clear where knowledge is concentrated and where important gaps in understanding remain, either within or among elements. This approach can also be broadened to gauge and communicate the types of knowledge available in different ecosystems and to compare the emphasis of ongoing investigations or modeling.

The four programs we have compared focused mainly on composition (GoMx) or on a combination of composition and structure; GBRSB had the greatest detail on structural elements but was restricted to the seabed (Table 3). In all four of these systems and studies, there is a relative paucity of investigation on functional elements of biodiversity, when compared with compositional and structural elements. This is symptomatic of the current state of the science. The functional elements that were addressed primarily focused on human-induced influences (fishing, eutrophication, and management interventions) and natural influences (such as sediment characteristics and hydrographic conditions). We note that globally, numerous studies have looked at human impacts on biodiversity composition and structure [161], [162] and the response of communities when human disturbance is reduced [3], [163], [164]. These represent important management considerations, but do not address many other internal structuring forces such as natural variations in populations. The B-HMAP program examined biological feedbacks on biodiversity, but only within a limited trophic context. At this time, natural functional elements probably represent the least understood drivers of biodiversity composition and structure. Often, tactical management questions involving processes can be addressed by surrogate indicators from composition and structure. Hence, management funding agencies tend to see studies of marine processes as pure science rather than a tactical imperative, and thus do not fund them. This ultimately carries a risk, because surrogates provide clues but do not, of themselves, provide for understanding of processes, and this is where basic science and issue-driven science can meet.

### Links to EBM

Ecosystem-based management for the ocean is a relatively new approach that has been growing over the last few decades [165]. Examples of fully implemented, comprehensive marine EBM are rare, however, probably because of incomplete scientific information and the difficulties inherent in implementing large-scale management strategies within complex natural and socioeconomic systems [12] (but see [166], [167] for examples of analyses to support implementation, and [12] for advanced examples). Although the Census of Marine Life was primarily a discovery program, there has been a strong demand for results from the Census to inform policy [168]. The four programs described here were conducted to organize and improve biodiversity knowledge so that it could be incorporated into regional management and policy, but only the GBRSB was designed by concurrent consultation between managers, scientists, and stakeholders; consequently it saw the most immediate application of the information gained. A key element of that consultation and uptake was the establishment of a Steering Committee with management and industry stakeholders to oversee the project.

Substantial investment in understanding one or more biodiversity element(s) will allow issues that come up over time to be addressed in a timely and more integrative fashion. For example, the detailed maps of composition and structure of seabed assemblages developed by the GBRSB have been applied to questions beyond the original issues that prompted the research. It could be argued that decisions about fishing restrictions could have been made with a simpler approach to mapping habitats, but the extra effort invested in sampling biota and quantifying habitat-biodiversity relationships resulted in much more objective and quantitative assessments, with additional benefits to management and science, including methodological developments, biological discoveries, and ecological insights.

### Linking biodiversity to ecosystem function

Everywhere there is a need to understand and document the linkage between biodiversity and the functioning of ecosystems. This includes ecosystems' emergent properties (functional attributes, resiliency, adaptation) and the services they provide to humans (some readily quantifiable, some not). Attempts to test hypotheses and thereby “demonstrate” some of these relationships using experimental approaches have provided great insights but also revealed limitations due to simplification or assembly of experimental communities [169], [170]. Meta-analyses have confirmed important generalities from such studies [171], [172] but do not provide the type of quantitative information needed to guide difficult and specific management decisions in an era of increasing human demands on the environment. There is a need for comprehensive investigations that blend oceanographic, biodiversity, natural ecological, and “ecosystem services” investigations to devise (and crucially, for society and managers to understand, accept, and adopt) truly sustainable practices in different types of habitats. Investigations should use theoretical, modeling, field measurement, disturbance/recovery, and environmental trajectory approaches linked to management scenarios. Managers and scientists must build in realistic expectations of time and resources for such large undertakings, including development (planning, funding, consulting with stakeholders), implementation (research, data entry, and analyses), communicating results (reports, scientific publications, presentations, release of databases), and, ultimately evaluating the results of management actions.

Understanding the function of biodiversity is important not only to marine science and management, but also to society. If biodiversity is to be conserved, it will require public support. Second, diversity-function relationships must be understood in order to set management objectives, which ultimately must include the maintenance of productive and resilient ecosystems. Third, we must be able to assess our success or failure. How do we achieve this when both human and natural systems are dynamic? Since perturbations from both sources characterize the past and the future of marine systems, there can be no single or simple biodiversity-function “state” that is the target. How can we distinguish between acceptable “change” and unacceptable “degradation”? Given the degraded state of many coastal and shelf ecosystems [2]–[4], we believe that knowledge of historical states and dynamic change are important. Monitoring and assessment can be used to detect change and assess management practices but are not sufficient for successful EBM; integration of knowledge into system understanding is needed. Modeling frameworks are particularly useful, including modeling of the biophysical system, the dynamics of human use, and the management process [167].

## Supporting Information

Text S1 (DOC)
