# Supplementary material for: Four Regional Marine Biodiversity Studies: Approaches and Contributions to Ecosystem-Based Management
Source: PLoS One. 2011 Apr 29;6(4):e18997. doi: 10.1371/journal.pone.0018997 (PMC3084743; doi:10.1371/journal.pone.0018997)
Supplement: Text S1 — (DOC) [file pone.0018997.s001.doc]

Text S1.

Ramsar Convention (1971), Bern Convention (1979), European Union Birds Directive (1979), Bonn Convention (1979) Agreement on Conservation of Small Cetaceans of the Baltic and North Seas (1991), European Union Common Fisheries Policy (1998), European Union Habitats Directive (1992), United Nations Convention on Biological Diversity (1992), European Union Water Framework Directive (2000), European Union Maritime Policy (2007), and European Union Marine Strategy Framework Directive (2008)
